# DEVELOPMENT AND VALIDATION OF THE LEISURE EUSTRESS-DISTRESS SCALE

**DOI:** 10.1093/geroni/igac059.1880

**Published:** 2022-12-20

**Authors:** Jaesung An, Laura Payne, Toni Liechty

**Affiliations:** California State University East Bay, Cupertino, California, United States; University of Illinois Urbana-Champaign, Champaign, Illinois, United States; University of Illinois Urbana-Champaign, Champaign, Illinois, United States

## Abstract

Encountering stressors is a part of everyday life and can even occur when people engage in leisure activities. This is more prevalent among older adults due to the onset of chronic condition(s), lack of resources, and social isolation. Therefore, leisure related stressors could provoke complex emotions derived by positive and/or negative responses to stressors before, during, and after older adults engage in leisure activities. Different from the concept of leisure constraints, which emphasizes causes of stress that prevents leisure engagement in the first place, the concepts of leisure eustress and distress are associated with the emotional experience of stress in the context of leisure. Despite the presence of leisure-based eustress and/or distress among older adults, there is no measurement scale that assesses this phenomenon. Therefore, the purpose of this study was to develop and validate a leisure eustress-distress scale (LEDS). Phase one of this study included in-depth interviews, expert panelists review, and a pilot study to develop and refine items. In the second phase, a target study was conducted to examine the factor structure using exploratory and confirmatory factor analysis. For validity, LEDS showed partial support for construct and criterion validity. Strategies to strengthen the scale’s validity will be discussed as well as future recommendations and practical implications of the LEDS.

